# A High-Temperature MEMS Surface Fence for Wall-Shear-Stress Measurement in Scramjet Flow

**DOI:** 10.3390/s17102412

**Published:** 2017-10-22

**Authors:** Chengyu Ma, Binghe Ma, Jinjun Deng, Weizheng Yuan, Zitong Zhou, Han Zhang

**Affiliations:** Key Laboratory of Micro/Nano Systems for Aerospace, Ministry of Education, Northwestern Polytechnical University, Xi’an 710072, China; macy@mail.nwpu.edu.cn (C.M.); dengjj@nwpu.edu.cn (J.D.); yuanwz@nwpu.edu.cn (W.Y.); rudolphzzt@mail.nwpu.edu.cn (Z.Z.); zhanghan016@mail.nwpu.edu.cn (H.Z.)

**Keywords:** surface fence, wall shear stress, scramjet flow, high temperature

## Abstract

A new variant of MEMS surface fence is proposed for shear-stress estimation under high-speed, high-temperature flow conditions. Investigation of high-temperature resistance including heat-resistant mechanism and process, in conjunction with high-temperature packaging design, enable the sensor to be used in environment up to 400 °C. The packaged sensor is calibrated over a range of ~65 Pa and then used to examine the development of the transient flow of the scramjet ignition process (Mach 2 airflow, stagnation pressure, and a temperature of 0.8 MPa and 950 K, respectively). The results show that the sensor is able to detect the transient flow conditions of the scramjet ignition process including shock impact, flow correction, steady state, and hydrogen off.

## 1. Introduction

In scramjet flow, the drag contribution caused by wall shear stress accounts for more than half of the total drag force. Therefore, accurate estimation of the wall shear stress is very important for evaluating the engine performance. However, the harsh environment makes large majority of shear-stress sensors difficult to be used normally. For example, MEMS floating-element sensors based on a capacitance sensing design present high performance in dynamic measurements [1]. However, the comb-fingers capacitors are susceptible to contamination particles, resulting in output errors or even a sensor failure. The micro-pillar shear-stress sensors [2,3], surface stress sensitive films [4], and flexible thermal sensor arrays [5,6] can be used upon curved surface for the distributive measurement. Nevertheless, their materials’ mechanical properties or sensitivities are compromised at high temperature. MEMS surface fence is a piezoresistive cantilever shear-stress sensor [7,8,9,10,11,12,13]. Deflection of the sensitive cantilever, proportional to wall shear stress, can be directly measured by stress-sensitive resistors placed in stress concentration areas. Unfortunately, these junction-isolated piezoresistors severely restrict applications in high-temperature environments. At present, bulky friction balance based on strain gauge is dominant in shear-stress estimation under sustained high-enthalpy flow conditions [14,15,16,17]. For the large-diameter floating head, which deflects under shear loads, obtaining a quick response and shear-stress fluctuations is difficult.

This paper presents the design, fabrication, calibration, and testing of a MEMS shear-stress sensor. This new sensor is expected to be used in high-speed, high-temperature flow conditions. The mechanism of the high-temperature heavily doped polysilicon resistance is studied. The operating temperature limit of the sensor is greatly enhanced (compared to our previous research). High-temperature packaging is specially designed to make the sensor applicable to the high-enthalpy flow. Finally, the sensor is used to examine the transient flow conditions of the scramjet ignition process, and the measured wall shear value agrees with the Van Driest estimation.

## 2. Design and Fabrication 

### 2.1. Theoretical Basis of High-Temperature Resistance

Polycrystalline silicon is composed of grains of monocrystalline silicon and grain boundaries. Thus, its material resistivity can be expressed as

(1) $$\rho =\rho_{c}\times 2W/L+\rho_{d}\times (1-2W/L)$$

where $\rho_{c}$ and $\rho_{d}$ are the resistivity of monocrystalline silicon and grain boundary, respectively. The dissipation region width at the grain boundary and the grain size are denoted by *W* and *L*, respectively. In general, the left side of Equation (1) is dominated by $\rho_{c}$. This leads to similar heat-resistant abilities for mono- and poly-crystalline silicon resistors (in practice, below 250 °C), which is just the limitation in the development of high-temperature Si-based piezoresistive devices.

Things change in the heavily doped polycrystalline silicon with a doping concentration of impurity higher than the threshold value, 1 × 10^20^ cm^−3^. The resistors of this material will a have current modification property [18] when the forced current density is higher than the other threshold value, which is about 1 × 10^6^ A/cm^2^. In these cases, ρ is completely dominated by $\rho_{d}$, so the physical and structural changes at the grain boundaries, which are attributed to Joule heating due to the large current density, can significantly enhance the thermal stability of the material. As a result, a higher operating temperature limit can be achieved.

### 2.2. Analysis of Key Process Parameters

The current density at normal operating conditions is actually much lower than 1 × 10^6^ A/cm^2^. However, the allowable forced current density should be maximized for better high-temperature stability. A simplified model of the resistor structure can be regarded as a rectangular cube with a cross section of *h* × *w* and a length of *l*. Thus, the current density along the length direction, *J*, can be defined as

(2) $$J=\frac{U/R}{w\cdot h}$$

where *w* is the width of the resistor, and *h* is the polysilicon deposition thickness. The piezoresistive resistance, *R*, can be represented by

(3) $$R=\frac{\rho }{t}\cdot \frac{l}{w}$$

where *t* represents the junction depth. Thus, Equations (2) and (3) give

(4) $$J=\frac{U\cdot t}{\rho \cdot l\cdot h}$$

where *U* and *l*, in most cases, determined by the DC-regulated power supply and mask, respectively, are supposed to be constant. To maximize Equation (4), the two following conditions should be satisfied.

- The junction depth of the resistor is equal to the polysilicon deposition thickness due to the relationship of t≤h.
- The resistivity of the polycrystalline silicon is less than 0.005 Ω·cm, corresponding to the doping concentration greater than 1 × 10^20^ cm^−3^.

### 2.3. Fabrication Process

**Figure 1:**
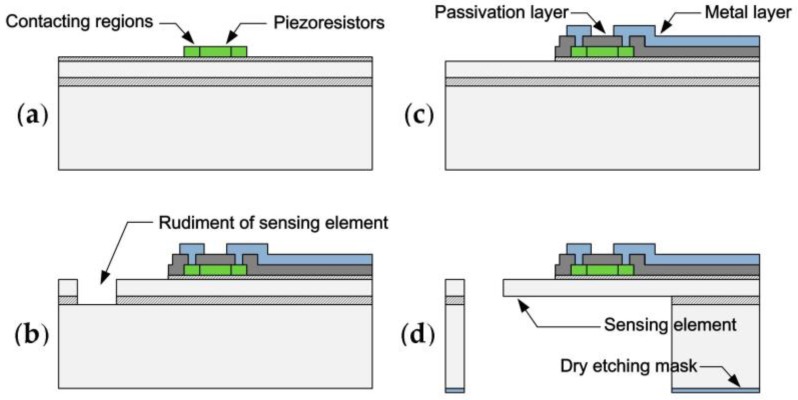
Fabrication process of the high-temperature MEMS surface fence. (**a**) The heavily doped polysilicon piezoresistors are fabricated. (**b**) The metal layers above the passivation layers are sputtered and patterned for better wire-bonding reliability at high temperature. (**c**) The rudiment of the sensing element is patterned by the ICP process. (**d**) Back-etching helps release the sensor.

The sensor is manufactured using a combination of bulk- and surface-micromachining with five lithography masks. 4” SOI wafers are used, and the thicknesses of the device layer and buried oxide (BOX) are 35 μm and 0.5 μm, respectively. At the beginning, the electrical isolation (200 nm SiO_2_) is thermally grown on the substrate. Then, the polycrystalline silicon layer (thickness of 200 nm), deposited by low-pressure chemical vapor deposition, is patterned and etched into mutually independent structures via ion reactive etching (RIE). Rapid thermal annealing (RTA, 850 °C for 30 min) before ion implantation is specially carried out for grain growth and recrystallization. Doping parameters of the patterned polysilicon film are particularly designed (Boron, 20 keV and 8 × 10^15^ cm^−^^2^ dose), so the electrical trimming property that exists for the impurity concentration and the applied current density is obtained (Figure 1a). The passivation layer (250 nm SiO_2_, 50 nm Si_3_N_4_) is deposited and removed on the cantilever structure, except for the regions covering the piezoresistors. The contact areas are etched by the RIE and the buffered oxide etch (BOE), and then Ti/Pt/Au, as the metal layers are sputtered and patterned for better wire-bonding reliability at high temperature (Figure 1b). By applying a current pulse (density higher than a certain threshold value) on these polysilicon resistors, the physical and structural changes at the grain boundaries occur. This modification process, similar to an electrical aging process, can significantly enhance the thermoelectric properties of the fabricated bridge-arm resistance. Consequently, the range of the sensor operational temperature is promoted.

In structure manufacturing, the rudiment of the sensing element is patterned by the inductively coupled plasma (ICP) process. The BOX serves as an etch stop layer, and a Bosch process with short etch cycles allows a smooth perpendicular fence edge. The naked BOX is removed ahead of the back-etching process to simplify the release process (Figure 1c). The bulk silicon underneath the fence is etched by ICP (the patterned Al-layer is used as the etching mask), and when etching stops at the remaining BOX, the sensing element structure is directly released at the same time (Figure 1d). 

Note that conventional ICP with a Bosch process should not be used in etching the polycrystalline silicon. The main reason is that the deposition of fluorinated carbon polymer caused by a time-multiplexed etch-passivate process may lead to failure of the ohmic contact.

### 2.4. Sensor Structure and Operating Theory

**Figure 2:**
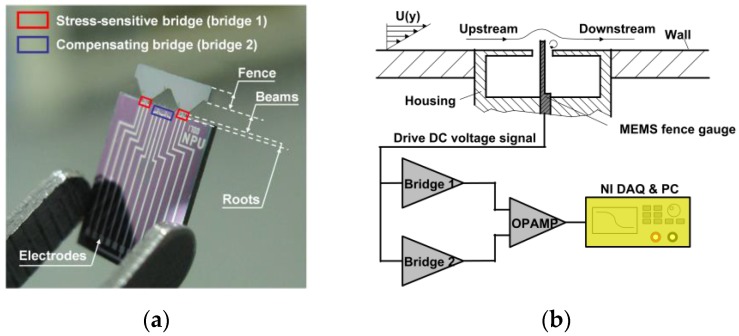
(**a**) The MEMS surface fence and its structure. (**b**) The working principle diagram of the shear-stress sensor and its simplified measurement setup.

The entire sensor is a monolithic silicon device that consists of a silicon body and an elastic sensing element, as shown in Figure 2a. The silicon body provides contacting and safe handling during packaging, while the elastic sensing element is used to sense the pressure difference. The elastic sensing element has three parts, namely fence, trapezoidal beams, and roots. The fence can work in the viscous sublayer, and a fluid flowing towards this structure would generate a pressure difference that depends on the fence dimensions and wall shear stress only. The resulting bending stress, which is also proportional to the wall shear stress, can be directly measured by a Wheatstone bridge with four integrated resistors which are located on the roots.

A compensating bridge (denoted as Bridge 2) that is free from stress interference (only sensitive to temperature) was also designed. It is connected to the interface circuit for eliminating the sensor thermal drift. A drive DC voltage signal is applied on the electrodes, and the differential amplified output voltages can be recorded by an NI DAQ & PC system, as shown in Figure 2b.

### 2.5. High-Temperature Packaging

**Figure 3:**
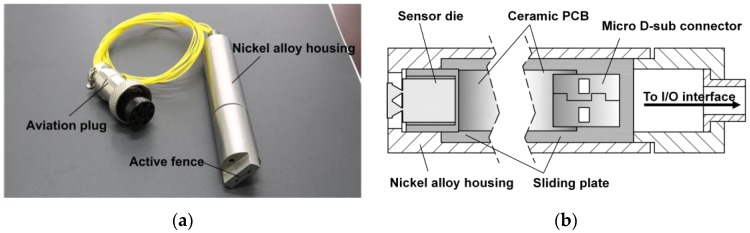
(**a**) Photograph of the packaged surface fence sensor; (**b**) A length-wise cross-section illustration of the shear-stress sensor package designed for use in high-temperature environment.

A high-temperature package was designed, as shown in Figure 3. Its overall dimension was 69 mm in axial length and 15 mm in diameter. Several types of materials in regard to thermal conductivity are adopted. The outer housing made of nickel alloy was plasma-sprayed with the thermal barrier coating (TBC). A sliding plate made of stainless steel with a polishing process was used for accurate installation for the micro fence sensor. The chip wiring was routed out through a ceramic PCB to a micro USB connector, which allows for plug and play. This terminal was then wired to an aviation plug, which was used as the input/output interface. 

Note that part of the fence structure could protrude from a small gap in the top of the package housing, which would be flush-mounted on the wall. The fence height above the wall (active fence height) could be adjusted for the different depths of the viscous sublayer. 

## 3. Sensor Characterization

### 3.1. Temperature Tests

The fabricated device was tested by heating the sensor die in a vacuum oven with air atmosphere and recording the non-amplified sensor output signals. All measurement conditions were held at a steady state for approximately 15 min before moving to the next temperature. High-temperature cement was used to fix the sensor to a custom-made ceramic printed circuit board (PCB). Wire bonding ensures stable electrical interconnection between the sensor electrodes and one end of the PCB in which the opposite end was routed out through mica wire to the data acquisition system. 

**Figure 4:**
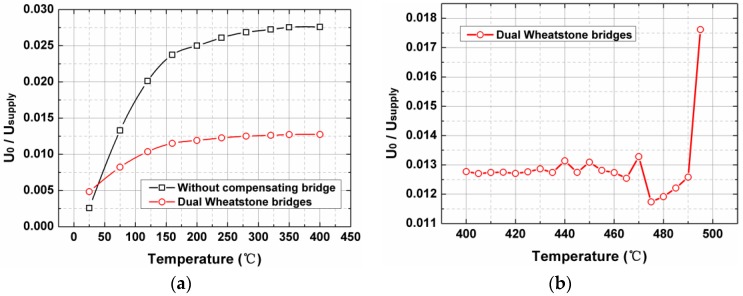
(**a**) Normalized residual temperature drift of the high-temperature MEMS surface fence. (**b**) The abnormal sensor outputs at higher temperature.

In this case, the residual temperature drift of the sensor was determined, as shown in Figure 4a. A four-order polynomial was suitable while fitting the zero-drift curves. The sensor offset voltages started with a monotonically increasing trend but gradually leveled off after 250 °C, and even if the temperature rose to 400 °C, the outputs still exhibited good stability. It could also be seen from the diagram that the dual Wheatstone bridges design significantly inhibited the temperature dependence of the offset voltage of the sensor, compared with the outputs without a compensating bridge. The sensor outputs did not remain stable as excepted when the temperature rose above 420 °C, as shown in Figure 4b. This problem is probably derived from the deterioration of the electrical connection performance and the precipitation of the implanted ions. Impurity-doped polysilicon has conductivity, and these ions are somewhat similar to the ferroelectric domains of PZT. However, different from PZT’s transition phase change and depolarization [19] caused by high temperature, the main reason for performance deterioration of the fence sensor at higher temperature is the transition from the semiconductor to the intrinsic semiconductor. With the increase in temperature, the intrinsic carrier concentration gradually increased. When the intrinsic carrier concentration approached the impurity carrier concentration, the electrical performance of the sensor became unstable. As the temperature continued to rise, the semiconductor became an intrinsic semiconductor, and at the same time, the sensor became invalid. Note that the sensor involved in the tests has not yet been packaged, so the obtained maximum operating temperature of 400 °C only refers to that of the sensor itself.

### 3.2. Wall-Shear-Stress Calibration

Test facilities are located at China Aerodynamics Research and Development Center (CARDC). The wind tunnel consists of a high-pressure gas source, a pressure regulator, a settling chamber, a contraction section, and a measurement section (200 mm × 15 mm) with a length of approximately 2.0 m. The boundary layer is tripped by strips of coarse-grained sandpaper 40 cm downstream from the contraction. The fence sensor and a reference Preston tube are flush mounted on the opposite sides of the wall at the same axial location for the same wall shear stress, and of course, the classical calibrations of Patel [20] are used. The sensor output signals are amplified 10-fold with AD8221 precision instrumentation amplifiers to improve the signal-to-noise ratio (SNR). Data samples were taken by a 16 bit DAQ (NI USB-6120) for high resolution. 

**Figure 5:**
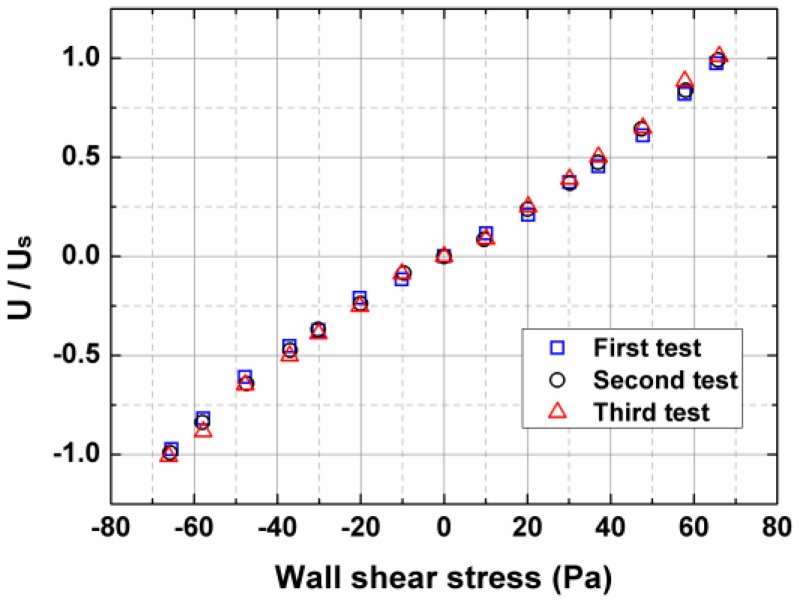
Plot of the normalized changes in output voltages with respect to the varying wall shear stress.

The output signals of the fence sensor, as a function of the wall shear stress, are plotted in Figure 5. The Mach range of the flow is 0.3~0.65, and the corresponding Reynolds number ranges from 7.3 × 10^4^ to 2.6 × 10^5^. Good agreement can be observed in these three repeatability tests. However, because of the possible influence of the turbulence structures, the scatter in the data occurred at higher Reynolds numbers. The calibration curve is not a straight line, and this non-linearity response has been noted before [21]. The active fence height was specially selected, which ensured that the fence lay within the viscous layer. Note that the negative wall-shear-stress values are actually obtained by rotating the sensor 180 degrees.

## 4. Measurement in Scramjet Flow

### 4.1. Test Apparatus

Under scramjet flow conditions, harsh environments increase the difficulty of measuring even the most basic aerodynamic characteristics. Thus, it is an exciting thing to try to understand the development of the transient flow in a scramjet ignition process by detecting the surface shear stress.

**Figure 6:**
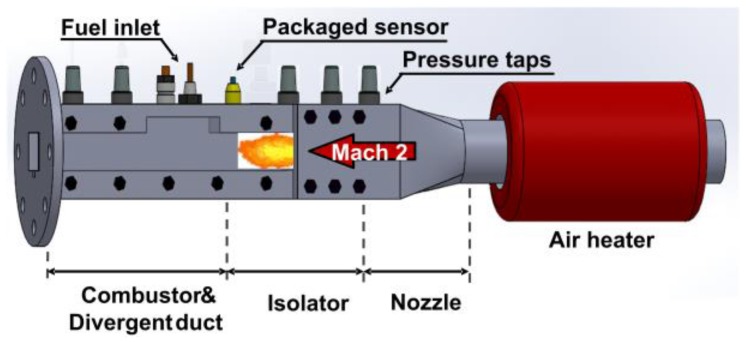
Schematic diagram of the main testing structure of a small-scale, high-unit Reynolds number, direct-impulse combustion tunnel facility.

At CARDC, the small-scale, high-unit Reynolds number, direct-impulse combustion tunnel facility is designed to supply supersonic combustion flows. Its main testing structure is shown in Figure 6. O_2_ + N_2_ and H_2_ + N_2_ are fed into a combustion heater to generate a high-enthalpy airflow supplied into the combustor. The vitiated air consisting of N_2_, O_2_, and H_2_O (their mole ratio is 67%, 21%, and 12%) is accelerated by the two-dimensional (2-D) nozzle to a nominal Mach 2, and the unit Reynolds number is 2.1 × 10^7^ m^−1^. The total pressure, the wall pressure, and stagnation temperature at the nozzle exit are 0.8 MPa, 100 kPa, and 950 K respectively. The specific heat ratio of the test gas in the core flow is 1.37, and the ratio of the temperature near the wall to the static temperature is about 1.

### 4.2. Initial Test and Analysis

In these measurements, the fence sensor is located in the downstream of the isolator entrance on centerline, and the distance from the entrance of the isolator section is 300 mm. The boundary layer is turbulent and quasi-2D, which is confirmed by boundary layer surveys. Thus, the calibration of the fence sensor should be carried out in the full development turbulent flow, as we have already done. Because the flow is almost free of shock wave interference and obeys the law of the wall, the fence sensor is applicable to this flow conditions. 

**Figure 7:**
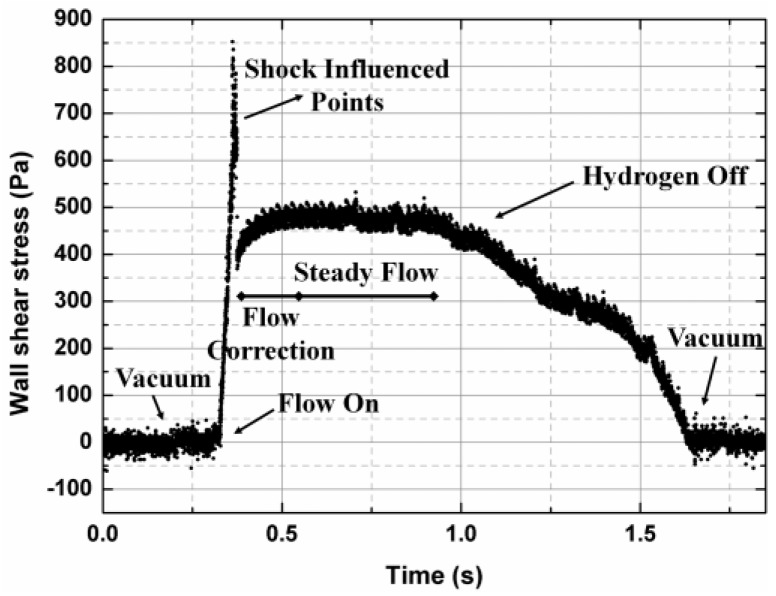
The measured wall shear values by the high-temperature MEMS surface fence located in the downstream of the isolator entrance.

Figure 7 shows the one time trace of the experimental shear-stress data. The variation trend of the curve still remains fairly similar to that of the wall pressure at the sensor nearby. At the beginning, under no flow condition, the fence sensor remained at zero output. When the vitiated air started the tunnel, the wall shear stress suddenly increased. At this moment, a few data points were recorded that are apparently “outliers” due to shock or complex flow interaction. Subsequently, flow correction occurred over a range from ~0.4 to 0.6 s. At this stage, the wall shear values gradually increased and then leveled off. The duration of the steady flow condition was about 0.4 s, and the supplied hydrogen was then exhausted, as demonstrated by the slow drop in the wall-shear-stress experimental data.

The average wall-shear value under stable condition was measured at approximately 486 Pa for skin friction coefficients of 0.0018. This demonstrates good agreement with the analytical estimation of wall shear stress, which was implemented through the well-accepted Van Driest II model [22]. Overall, the shear-stress sensor experimental data indicates the development of the transient flow conditions of the scramjet ignition operation, which includes shock impact, flow correction, steady state, and hydrogen off.

## 5. Conclusions

The presented sensor offers new possibilities of wall-shear-stress measurements in high-speed, high-temperature scramjet flow. The heat-resistant mechanisms, key process parameters, and high-temperature packaging indicate that the sensor can be used at higher temperatures. The calibrated fence sensor was successfully used to determine the development of the transient flow in a scramjet ignition process by detecting the surface shear stress, and the measured values demonstrate good agreement with the analytical estimation of wall shear stress. The significant contribution or innovation of this article consists in the following.

(1) A breakthrough has been made in the operating temperature of the MEMS surface fence shear-stress sensor. Several studies including deposition, ion implantation, heat treatment, and current trimming have to be made to realize a higher operating temperature of 400 °C (the temperature tolerance of the sensor chip itself, which is independent of the packaging structure). The corresponding manufacturing technology of the high-temperature polysilicon piezoresistors we proposed is universal. It can also help other Si-based piezoresistive sensors improving their upper limit of operating temperature.
(2) The wall shear stress in transient scramjet flow has, for the first time, been achieved by a MEMS shear-stress sensor. Currently, the bulky custom-made high-temperature friction balance is the only device capable of measuring the skin-friction under high-enthalpy flow conditions. However, it features a low response frequency (≤200 Hz) and poor spatial resolution (in the order of centimeters), which is not applicable to the transient flow measurement. This will lead to a problem: once the scramjet engine fails to start (as we know, the primary problem in supersonic combustion is how to ignite), the bulky balance will be unable to detect the instantaneous friction values. A lack of the measured skin-friction information will lead to a gap in the understanding of boundary layer development, which is detrimental in solving the problem. It can be seen that our research has great scientific significance and engineering value for scholars who are engaged in engine research.

For applications at higher temperature (for example, combustion chamber), the presented sensor may be inadequate. However, new designs are currently under preparation: a MEMS surface fence based on optical fiber F-P cavity technology will be proposed. The measuring principle of this sensor is basically the same as that of the traditional surface fence. Benefitting from new material and the optical inspection method, measurements at even higher temperatures will be achieved.
